# Rapid microwave activation of waste palm into hierarchical porous carbons for supercapacitors using biochars from different carbonization temperatures as catalysts†

**DOI:** 10.1039/c9ra03031j

**Published:** 2019-06-20

**Authors:** Chaozheng Liu, Weimin Chen, Meichun Li, Shu Hong, Wanzhao Li, Mingzhu Pan, Qinglin Wu, Changtong Mei

**Affiliations:** College of Materials Science and Engineering, Nanjing Forestry University No. 159 Longpan Road Nanjing 210037 China mei@njfu.edu.cn +86-25-5427742; Jiangsu Engineering Research Center of Fast-growing Trees and Agri-fiber Materials Nanjing 210037 China; School of Renewable Natural Resources, Louisiana State University Baton Rouge LA 70803 USA

## Abstract

A rapid, simple and cost-effective approach to prepare hierarchical porous carbons (PCs) for supercapacitors is reported by microwave activation of abundant and low-cost waste palm, biochar (BC) and KOH. BCs from waste palm at different carbonization temperatures (300–700 °C), as catalysts and microwave receptors, were used here for the first time to facilitate the conversion of waste palm into hierarchical PCs. As a result, the high-graphitization PC obtained at a BC carbonization temperature of 300 °C (PC-300) possessed a high surface area (1755 m^2^ g^−1^), a high pore volume (0.942 cm^3^ g^−1^) and a moderate mesoporosity (37.79%). Besides their high-graphitization and hierarchical porous structure, the oxygen doping in PC-300 can also promote the rapid transport of electrolyte ions. The symmetric supercapacitor based on the PC-300 even in PVA/LiCl gel electrolyte exhibited a high specific capacitance of 164.8 F g^−1^ at a current density of 0.5 A g^−1^ and retained a specific capacitance of 121.3 F g^−1^ at 10 A g^−1^, demonstrating a superior rate capacity of 73.6%. Additionally, the PC-300 supercapacitor delivered a high energy density of 14.6 W h kg^−1^ at a power density of 398.9 W kg^−1^ and maintained an energy density of 10.8 W h kg^−1^ at a high power density of 8016.5 W kg^−1^, as well as an excellent cycling stability after 2000 cycles with a capacitance retention of 92.06%.

## Abstract 2

A rapid, simple and cost-effective approach to prepare hierarchical porous carbons (PCs) for supercapacitors is reported by microwave activation of abundant and low-cost waste palm, biochar (BC) and KOH.

## Graphical abstract

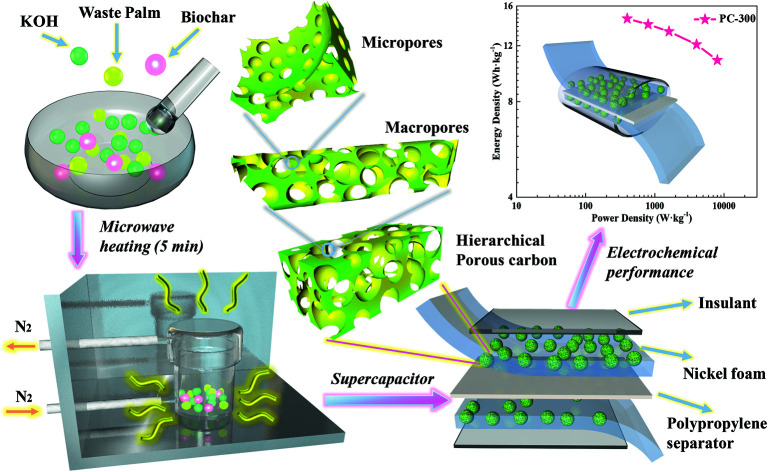

## Introduction

Supercapacitors, due to their high power density, rapid charge–discharge performance and excellent cycle stability, have emerged as a new type of environmentally friendly electrochemical energy storage system.^1–4^ In recent years, supercapacitors with biomass-based carbon materials (BCMs) as electrodes have attracted much attention.^5–8^ Not only the environmental friendliness and sustainability of biomass, but also their natural features have great potential as the precursors in the preparation of carbon materials for supercapacitors.^9^ Among them, palm is a low-cost and abundant agricultural waste, which has a high fixed carbon content to be well activated into BCMs.^10^ In addition, the interconnected porous structure of BCMs can facilitate the rapid transport of ions and doping heteroatoms into BCMs and can improve the pseudo-capacitance performance, which both enhance the electrochemical performance of the obtained supercapacitors.^9,11,12^ However, the synthesis of BCMs by conventional furnaces is costly, time-consuming and complex. This is because tubular furnace heating requires a long period of an inert atmosphere and forms a temperature gradient that could somehow affect the quality of the carbon materials.^13,14^ Therefore, it is urgent to find a simple, low-cost and fast method for the preparation of BCMs in supercapacitors.

Microwave heating is an effective alternative to conventional heating for activating biomass into carbon materials due to its less duration, smaller energy consumption and higher heating efficiency.^15–19^ Microwave-induced KOH activation is a common method to prepare PC, which is due to that KOH can be as an activator to enhance its porosity, especially the formation of micropores.^20^ In general, biomass is a poor microwave receptor and it cannot be heated to the temperature required for complete activation by microwave heating. However, carbon materials are good microwave absorbers, which can be mixed with the raw material to further promote microwave activation.^21–24^ Also, carbon materials are inexpensive, abundant and pyrolyzed without adding any other inorganic components. In addition, carbon residues from pyrolysis of organic materials are also good microwave absorbers.^25,26^ Hence here to use biochars from raw materials as catalysts and microwave receptors for facilitating the fast microwave conversion of waste palm into PCs in 5 min. This novel method can further reduce the reaction time and improve the efficiency, as well as avoid the use of chemical reagents and subsequent treatment.

As is well-known, carbon materials have the better electrical conductivity due to their higher graphitization and better crystalline structure. Also, the carbon materials treated at high temperatures are easier to form the crystalline structure of graphite and improve the graphitization degree.^27^ Ruiz *et al.* investigated the effects of thermal treatment (600 and 1000 °C) of activated carbon on the electrochemical behaviour in supercapacitors and demonstrated that the extra capacitance of the sample with high oxygen content has been proved to be due to the redox reactions.^28^ Tian *et al.* studied the influence of high temperature treatment of PC on the electrochemical performance in supercapacitor.^29^ The results showed that the improved capacitance of mesoporous carbon can be attributed to the expansion and reorganization of crystalline structure as well as the increase of mesoporous ratio. Thus, these factors, such as porosity, crystallinity or graphitization, which were affected by temperature and also influenced the specific capacitance. However, the ability of activated carbon to absorb microwave energy is affected by its pore size distribution and specific surface area.^30^ In addition, highly graphitized carbon materials can reflect part of the microwave radiation.^31^ Therefore, the BCs from different carbonization temperature, due to their different abilities to absorb microwaves, can be used as catalysts to prepare PCs from waste palm by microwave activation, and this has never been reported. In consideration of energy saving production, the carbonization temperature range was selected from 300 °C to 700 °C.

In this study, we synthesize the BCs from different carbonization temperatures as catalysts to facilitate the rapid microwave activation of waste palm into hierarchical PCs for supercapacitors. The abilities of BCs at different carbonization temperatures to absorb microwave were evaluated by simply analyzing their degree of graphitization and pore size distribution. The morphologies and microstructures of PCs were investigated by a series of characterizations and the electrochemical properties of PCs-based supercapacitors were further studied. By optimizing the carbonization temperature of BC to balance the pore structure and heteroatom doping of the PC, the obtained supercapacitor exhibits a high specific capacitance and superior rate performance. These exciting results have a huge potential for the rapid preparation of hierarchical PCs from waste palm for supercapacitors.

## Materials and methods

### Synthesis of PCs from waste palm

The waste palm (Nibong Tebal, Malaysia) was screened into a particle size of 20 mesh, and dried at 100 °C for 12 h. Then, the dried sample was placed into a porcelain boat located in a tube furnace for 1 h at different carbonization temperatures (300, 400, 500, 600, 700 °C) with a heating rate of 10 °C min^−1^ under a N_2_ atmosphere, denoted as BC-300, BC-400, BC-500, BC-600, BC-700. BCs have different abilities to absorb microwaves at different temperatures due to their crystallinity, graphitization and porosity, which were displayed by their XRD patterns and pore size distribution (Fig. S1†). The broad and weak peaks (the (002) and (100) reflection peaks) suggested a reduced degree of crystallinity and graphitization.^32,33^ Bake in a 100 degree oven for 12 hours 2.0 g of waste palm, 1.0 g of BC and 9.0 g of KOH were mixed together in a mortar, and the mixture was then heated in a microwave oven with the irradiation time of 5 min and the power of 700 W. The collected sample was repeatedly washed with 1 M diluted HCl and distilled water until the pH of the filtrate was 7.0, and then dried in an oven at 100 °C for 12 h. The obtained samples were denoted as PC-*x*, where *x* represented the carbonization temperature of BC. However, the PC was prepared by mixing with waste palm and KOH in a ratio of 1 : 3 without BC through microwave activation in 5 min and we found the activation was incomplete, so we didn't discuss it.

### Material characterization

The microstructure and morphology of the PCs were observed on field-emission scanning electron microscopy (FE-SEM, JSM-7600, operating at 10.0 kV) and transmission electron microscope (TEM, JEM-2100 with an accelerated voltage of 200 kV). The structural change, porosity and chemical composition of PCs were characterized by X-ray diffraction spectrometer (XRD, D8 Advance at a scan rate of 3° min^−1^), Raman spectrometer (DXR532, Thermo with *λ* is 532 nm), Micromeritics ASAP-2020 N_2_ adsorption/desorption analyzer and X-ray photoelectron spectra (XPS, AXIS UltraDLD, using Al Kα radiation).

### Electrochemical measurements

PCs, acetylene black and polytetrafluoroethylene binder (a weight ratio of 8 : 1 : 1) were well ground into slurry in ethanol that was coated onto half of a rectangular foamed nickel (2 × 1 cm^2^). The obtained nickel foams were dried at 100 °C for 8 h in a vacuum oven and then pressed at 10 MPa for 1 min, as electrode materials. The mass loading of the carbon materials in each electrode was about 5 mg cm^−1^. The symmetric supercapacitor was assembled by using polypropylene separator to separate two identical electrode materials. The PVA/LiCl gel was employed as electrolyte, which was prepared by adding 5 g of PVA and 10.6 g of LiCl into 50 mL of deionized water at 85 °C for 1 h under constant stirring.^34^ Cyclic voltammetry (CV), galvanostatic charge/discharge measurements (GCD) and electrochemical impedance spectroscopy (EIS) of the obtained supercapacitors were measured by an electrochemical workstation (Gamary Reference 600+). The sweep rates of the CV measurement were 5, 10, 20, 50, 100 mV s^−1^ and its voltage ranged from −0.4 V to 0.4 V. The current densities of the GCD test were 0.5, 1, 2, 5, 10 A g^−1^ and its voltage ranged from −0.4 V to 0.4 V. The frequency range of the EIS measurement was from 0.01 to 100 kHz. The specific capacitance (*C*, F g^−1^) based on the GCD, the energy density (*E*, W h kg^−1^) and the power density (*P*, W kg^−1^) were calculated by the following equations:^35,36^1
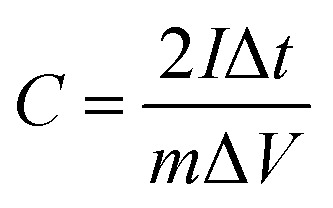
2
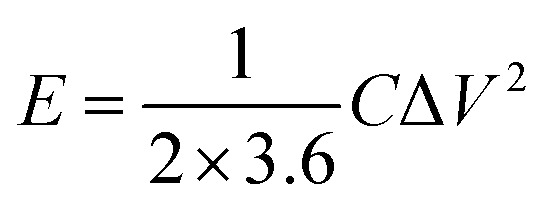
3
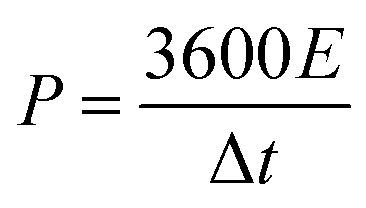
where *I* (A) is the discharge current, Δ*t* (s) is the discharge time, *m* (g) is the effective mass on a single electrode, and Δ*V* (V) is the voltage change excluding the voltage drop within Δ*t*, respectively.

## Results and discussion

### Characterization of the PC

**Scheme 1:**
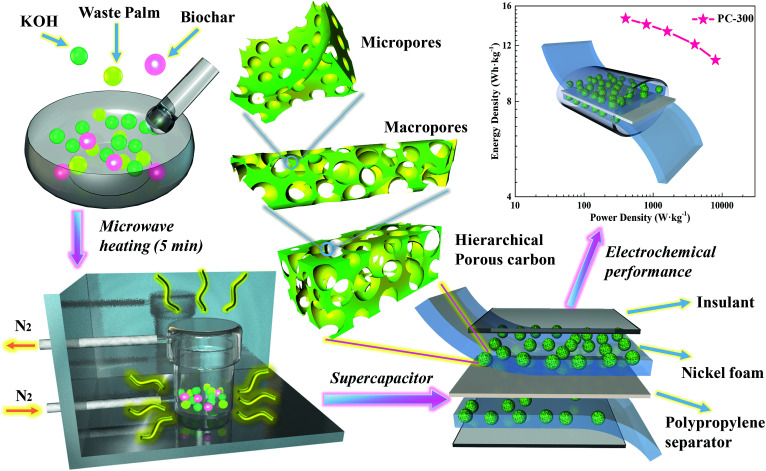
Schematic diagrams of the preparation for the symmetric two-electrodes supercapacitors.

**Fig. 1:**
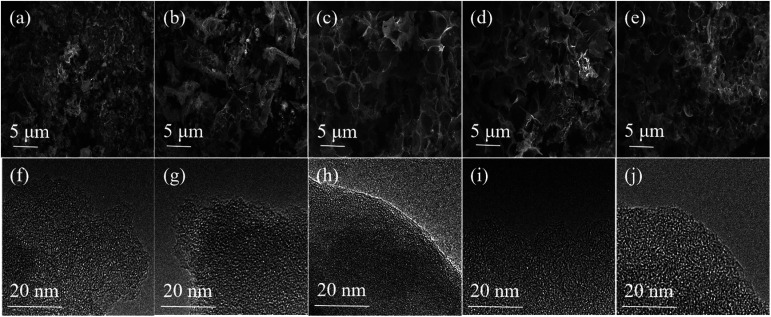
FE-SEM and TEM images of (a and f) the PC-300, (b and g) the PC-400, (c and h) the PC-500, (d and i) the PC-600 and (e and j) the PC-700 samples.

The schematic diagram of the symmetric two-electrode supercapacitor is shown in Scheme 1. The morphologies and microstructures of all samples were observed by FE-SEM and TEM. As shown in Fig. 1a–e, all samples were consisted of irregular macroporous and three-dimensional (3D) inter-connected structures. The macropores can shorten the ion transport paths, while 3D structures can further accelerate the diffusion of the electrolyte ions.^37,38^ The high resolution TEM images of all PCs exhibited mesoporous and microporous morphologies with highly structural disorder (Fig. 1f–j). In addition, the micropores can store electrolyte ions and the mesoporous can provide channels to promote ions diffusion.^39^ Thus, all samples showed porous structures after microwave activation, which is due to that KOH can react with carbon following 6KOH + 2C → 2K + 3H_2_ + 2K_2_CO_3_ and then decomposing K_2_CO_3_ with HCl washing.^5,40,41^ However, some large-sized macropores collapse due to the different microwave absorbencies of BCs at different carbonization temperatures. As a result, the PC-300 with hierarchical porous structure is significantly more conducive to the storage and transportation of electrolyte ions.

**Fig. 2:**
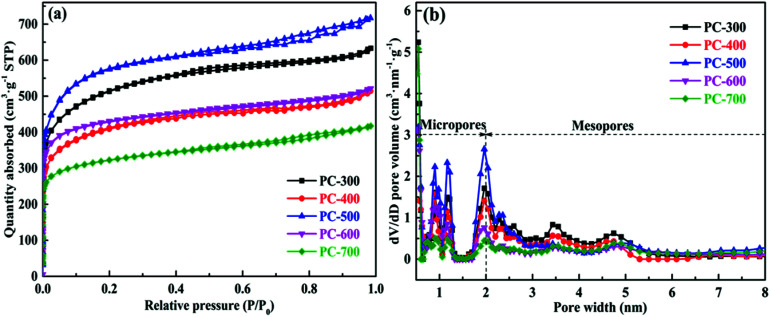
Nitrogen adsorption–desorption isotherms (a) and pore size distributions (b) of PCs.

The porous structures of all PCs were analyzed using nitrogen adsorption–desorption measurements. Fig. 2a showed the nitrogen adsorption–desorption isotherms of all PCs and each isotherm displayed a combined type I and type IV isotherm. The dramatic increase at a relatively low pressure (*P*/*P*_0_ < 0.2) and the slight increase at a high relative pressure (*P*/*P*_0_ = 0.9–1.0) in the isotherm indicate the presence of high-density micropores and macropores, respectively.^9,42^ Also, the hysteresis loop in the isotherm at a medium relative pressure (*P*/*P*_0_ = 0.4–0.8) reveals the presence of mesopores.^36^ Therefore, all PCs can be classified as hierarchical porous carbons, which were consistent with the results of the above micro-structural analysis. In addition, the pore size distribution curves of all PCs were shown in Fig. 2b. The pore size of all PCs mainly focused on 0.5–2 nm (micropores), together with mesopores of 2–6 nm. As well-demonstrated in these literatures,^43–45^ micropores can accumulate electrolyte ions, while mesopores and macropores can provide channels to transfer electrolyte ions to micropores and improve conduction efficiency. Therefore, the hierarchical PCs (containing macro-, meso- and micro-pores) have a great potential as an electrode material for supercapacitor. The PC-300 sample possessed a large surface area of 1755 m^2^ g^−1^, a high pore volume of 0.942 cm^3^ g^−1^ and a suitable mesoporousity of 37.9%, corresponding to the maximum specific capacitance in all samples. Moreover, the pore structure parameters of all PCs were presented in Table 1. The specific surface area, pore volume and mesoporousity of each sample are different, which indicated that the carbonization temperatures of BCs had an important influence on the development of pore structure of PCs.

**Table 1:** Structure parameters of carbon samples and their capacitive performance

Sample	*S*_BET_ a (m^2^ g^−1^)	*V*_t_ b (*V*_mic_^b^) (cm^3^ g^−1^)	Mesoporosity (%)	*D*_a_ c (nm)	Specific capacitance^d^ (F g^−1^)
PC-300	1755	0.942(0.586)	37.79	2.15	164.8
PC-400	1420	0.775(0.485)	37.42	2.18	122.9
PC-500	1944	1.067(0.673)	36.93	2.19	150.3
PC-600	1600	0.784(0.586)	25.26	1.96	148.1
PC-700	1196	0.633(0.421)	33.50	2.12	126.1

a Specific surface area (*S*_BET_) calculated from the Brunauer–Emmett–Teller (BET) method.

b Total pore volume (*V*_t_) calculated at *P*/*P*_0_ = 0.99 and micropore volume (*V*_mic_) calculated by *t*-plot method.

c Mean pore diameter calculated by the following equation: *D*_a_ = 4*V*_t_/*S*_BET_.

d Specific capacitance obtained at current density of 0.5 A g^−1^.

**Fig. 3:**
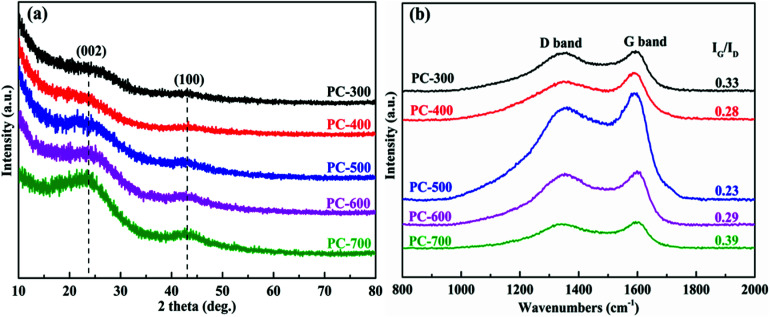
XRD patterns (a) and Raman spectra (b) of the PCs samples.

The structural changes of all PCs were investigated by XRD patterns (Fig. 3a). For PCs, two distinctive peaks positioned at approximately 23° and 43°, assigned to the (002) and (100) reflections of a graphitic-type lattice.^46^ The high intensity value in the small-angle region demonstrated large specific surface areas of PCs and the remarkable peak at 43° indicated the high condensation of interlayer that can effectively improve the electrical conductivity.^47^ In addition, the (002) peak became broad and weak as the carbonization temperature of BCs decreased, and almost disappeared in the PC-300 pattern. This result was due to the structural transformation of the PC from the turbostratic stacking layers to the spherical and ultra-thin stacking layers, corresponding to the change from the non-porous structure to the porous structure.^48^ The amorphous structures of all PCs were further investigated by Raman spectroscopy, as shown in Fig. 3b and S2.† There are two distinct characteristic peaks in all samples, corresponding to the D band (1350 cm^−1^) and the G band (1590 cm^−1^). The D band indicated the defects and disorder in PCs, while the G band demonstrated the graphitic order.^49^ Moreover, the integral intensity ratio of the G to D band (*I*_G_/*I*_D_) is used to estimate the level of graphitic structure in PCs. The *I*_G_/*I*_D_ of PC-300 (0.33) is relatively high, demonstrating superior graphitization, which promotes rapid diffusion of electrolyte ions to benefit the conductivity.^7,50^ It is noted that the *I*_G_/*I*_D_ ratio of PC-700 (0.39) was higher than that of PC-300, likely due to high graphitization of the BC-700, as revealed by the XRD results in Fig. S1a.†

**Fig. 4:**
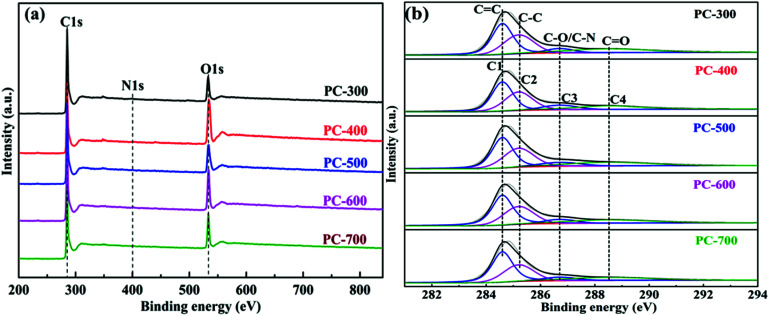
XPS spectra of all PCs (a) and high-resolution C1s (b) of the PCs samples.

The surface chemical compositions of the PCs samples were investigated by XPS. It is clear from the XPS survey (Fig. 4a) that all PCs contained three elements including carbon, oxygen and nitrogen. It has been proved in the literature that doping with nitrogen and oxygen can enhance the wettability of carbon materials to increase the accessible surface area for electrolyte ions and promote the pseudo-capacitance properties.^51^Table 2 listed the surface chemical compositions of all PCs obtained from XPS spectra. The high-resolution C1s spectra (Fig. 4b) of all PCs were resolved into four individual peaks, assigned to C=C (C1, 284.6 eV), C–C (C2, 285.2 eV), C–O or C–N (C3, 286.7 eV) and C=O (C4, 288.5 eV).^52^ The C1 peak corresponds to sp^2^ hybridized graphitic carbon and its content increased with the carbonization temperature of BCs increasing, indicating the formation of porous structure of carbon materials,^48^ which is consistent with the XRD results. The O1s spectra (Fig. S3†) were deconvoluted into four peaks at binding energies of 530.7 eV (O1, O–C=C–O), 531.8 eV (O2, C=O), 533.3 eV (O3, C–OH or C–O–C) and 535.2 eV (O4, O–C–O or –OH), respectively.^53,54^ It is noticed that the oxygen-containing functional group can be introduced into the PC surface due to its reaction with the defect site, which can improve the electrochemical properties of the PC-based supercapacitor.^55^ Moreover, the PC-300, due to its hierarchical porous structure with a high specific surface area, a high graphitization and a favorable doping of heteroatoms, has a great potential as high-performance electrode material for supercapacitor.

**Table 2:** Chemical composition of all PCs derived from XPS

Samples	XPS (at%)						
	C	O	N	C1	C2	C3	C4
PC-300	85.8	13.7	0.5	39.9	30.6	6.9	22.6
PC-400	66.0	33.4	0.6	40.1	35.9	7.4	16.6
PC-500	79.6	19.8	0.6	41.6	33.7	6.8	17.9
PC-600	76.2	23.2	0.6	44.9	30.8	7.5	16.8
PC-700	78.8	20.7	0.5	47.5	30.7	7.1	14.7

### Electrochemical property of the PC-based supercapacitor

**Fig. 5:**
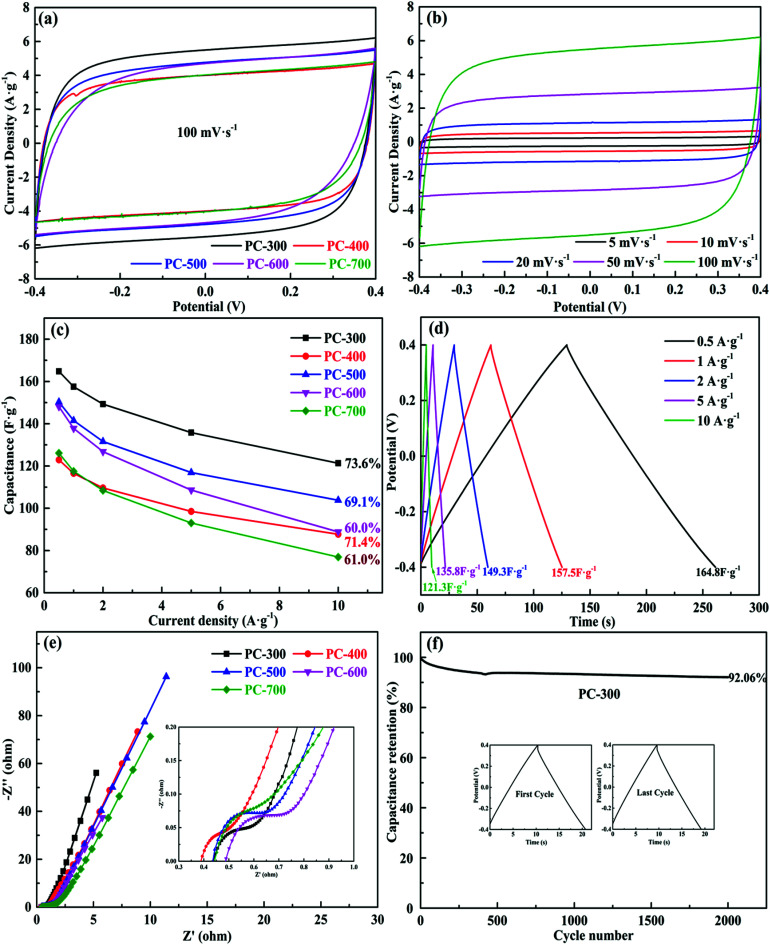
Electrochemical performance of all PCs-based supercapacitors using PVA/LiCl gel electrolyte. (a) CV curves at a scan rate of 100 mV s^−1^ in a voltage range from −0.4 V to 0.4 V, (b) CV curves of the PC-300 at different scan rates from 5 to 100 mV s^−1^, (c) specific capacitances at different current densities, (d) GCD curves of the PC-300 at various current densities from 0.5 to 10 A g^−1^, (e) Nyquist plots of the symmetric supercapacitors, and (f) cycling stability performance of the PC-300 at a current density of 5 A g^−1^.

The electrochemical properties of all the symmetrical supercapacitors were measured in PVA/LiCl gel electrolyte, using a two-electrode system. Fig. 5a compared the CV curves of all supercapacitors at a scan rate of 100 mV s^−1^ in a voltage range from −0.4 V to 0.4 V. The PC-300 showed the largest CV curve area in all samples, indicating the maximum specific capacitance. This result is consistent with the large specific surface area and well-developed pore distribution of PC-300 in BET analysis. Also, the CV curves of the PC-300 at different scan rates from 5 mV s^−1^ to 100 mV s^−1^ were displayed in Fig. 5b and other samples were showed in Fig. S4.† All the CV plots of the PC-300 exhibited quasi-rectangular shapes, demonstrating good electrochemical performance of this supercapacitor.^9^ The performance was further confirmed by the GCD measurement. The calculated specific capacitance of all supercapacitors at different current densities were revealed by Fig. 5c, and the GCD curves of the PC-300 at various current densities from 0.5 to 10 A g^−1^ showed highly symmetrical and linear (Fig. 5d), suggesting ideal capacitance performance. Moreover, Fig. S5† displayed the GCD curves of other samples. For all samples, the PC-300 achieved the highest specific capacitance of 164.8 F g^−1^ at 0.5 A g^−1^ even in the PVA/LiCl gel electrolyte and retained 121.3 F g^−1^ at 10 A g^−1^, showing the best rate capacity (73.6%). As the carbonization temperature of BC increased, the rate capacity of the obtained supercapacitor generally showed a decreasing trend, corresponding to the variation in mesoporosity of the electrode materials (*i.e.* PCs) in pore size analysis. Additionally, the IR drop of the PC-300 even at high current densities was small, indicating that its well-developed porous structure facilitated the rapid transport of electrolyte ions and supercapacitor have a small internal resistance.^56,57^ Furthermore, the Nyquist plots of all supercapacitors with inset showing the magnified high frequency region were displayed in Fig. 5e. In the high frequency region, all EIS curves exhibited short Warburg regions, assigned to short diffusion path of the electrolyte ions, which promoted fast transport of ions.^58^ Also, the equivalent series resistance (ESR) of all supercapacitors were small values (0.36–0.47 Ω), which is mainly attributed to the high content of oxygen heteroatom and hierarchical porous structure in PCs. In the low frequency region, the deviation in slope may be due to the contribution of pseudo-capacitance.^59^ The curve of the PC-300 was closer to *Y* axis than any others, which is due to its well-developed pore structure, demonstrating a high specific capacitance and an excellent rate capability. Fig. 5f and S6† showed the cycling stability performance of all the symmetric supercapacitors at a current density of 5 A g^−1^ after 2000 cycles. The PC-300 exhibited excellent cycling stability after 2000 cycles with a capacitance retention of 92.06%. Moreover, the charge and discharge curves of the first cycle was roughly the same as the last cycle.

**Fig. 6:**
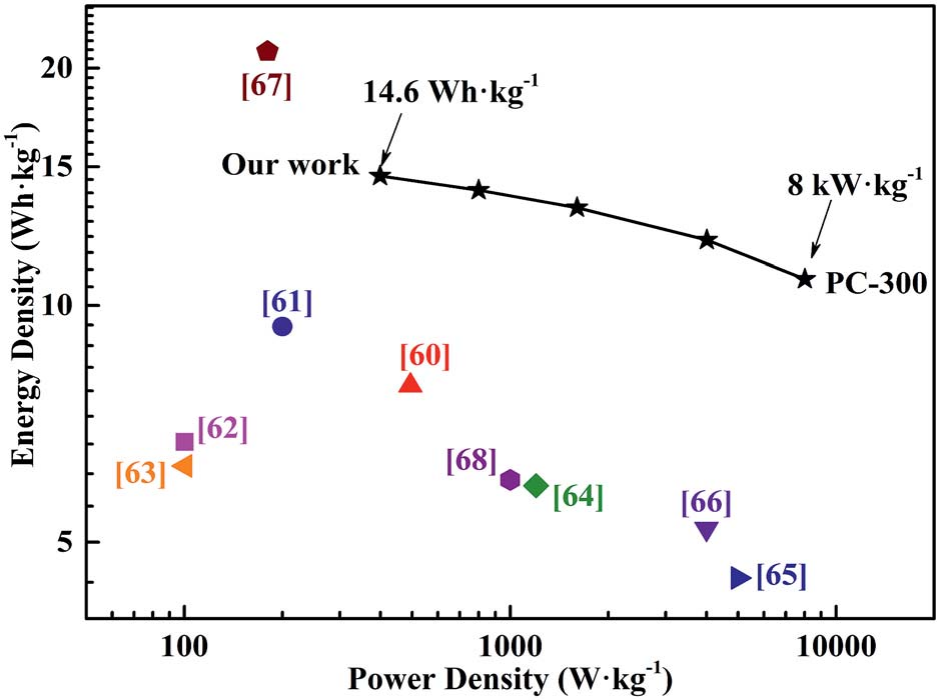
Ragone plots of the PC-300 supercapacitor and performance comparison with previously reported results.

The symmetric supercapacitor based on PC-300 electrodes exhibits a high energy density of 14.6 W h kg^−1^ at a power density of 398.9 W kg^−1^ and retains an energy density of 10.8 W h kg^−1^ at a high power density of 8016.5 W kg^−1^, showing a comparable or even superior to previously reported other carbonaceous materials based supercapacitors (Fig. 6), such as pectin biopolymer derived porous carbon (7.9 W h kg^−1^ at 494 W kg^−1^),^60^ carbon nanosheets from cornstalk (9.4 W h kg^−1^ at 200 W kg^−1^),^61^ highly porous graphitic biomass carbon (6.7 W h kg^−1^ at 100.2 W kg^−1^),^62^ porous carbon nanosheets from bagasse (6.25 W h kg^−1^ at 100.5 W kg^−1^),^63^ N-doped carbon nanofibers (5.9 W h kg^−1^ at 1200 W kg^−1^),^64^ 3D graphene hydrogen films (4.5 W h kg^−1^ at 5000 W kg^−1^),^65^ chemical modified graphene (5.2 W h kg^−1^ at 4000 W kg^−1^),^66^ carbon nanosheets from willow catkin (21 W h kg^−1^ at 180 W kg^−1^)^67^ and ordered mesoporous carbon (6 W h kg^−1^ at 1000 W kg^−1^).^68^ In addition, these results showed that microwave-induced KOH activation with BC as a catalyst is a good method to obtain PCs with hierarchical and well-developed porous structure, which was a superior electrode material for cost-effective and green supercapacitor with high energy-power density.

## Conclusions

In summary, we have developed a facile, green and cost-effective method for the rapid microwave synthesis of hierarchical PCs from waste palm using BCs from different carbonization temperatures as catalysts. The high-defect PC-300 possessed a high surface area (1755 m^2^ g^−1^), a high pore volume (0.942 cm^3^ g^−1^) and a moderate mesoporosity (37.79%). Also, the oxygen doping in PC-300 can promote the rapid transport of electrolyte ions. Due to the synergistic effect of porous structure and oxygen doping, the PC-300 supercapacitor exhibited a high specific capacitance of 164.8 F g^−1^ at 0.5 A g^−1^ and maintained 121.3 F g^−1^ at 10 A g^−1^, indicating a superior rate capacity (73.6%). Moreover, the PC-300 supercapacitor attained a high energy density of 14.6 W h kg^−1^ at a power density of 398.9 W kg^−1^ and maintained a energy density of 10.8 W h kg^−1^ at a high power density of 8016.5 W kg^−1^, as well as a superior cycling stability with a capacitance retention of 92.06% after 2000 cycles. These exciting results provide a great prospect for the preparation of hierarchical PCs from waste palm for supercapacitor applications.

## Conflicts of interest

There are no conflicts to declare.

## Supplementary Material

RA-009-C9RA03031J-s001
