# Supplementary material for: Rapid microwave activation of waste palm into hierarchical porous carbons for supercapacitors using biochars from different carbonization temperatures as catalysts
Source: RSC Adv. 2019 Jun 20;9(34):19441–9. doi: 10.1039/c9ra03031j (PMC9065327; doi:10.1039/c9ra03031j)
Supplement: RA-009-C9RA03031J-s001 [file RA-009-C9RA03031J-s001.pdf]

Electronic Supplementary Material (ESI) for RSC Advances.
This journal is © The Royal Society of Chemistry 2019

Supplementary Materials

Rapid microwave activation of waste palm into hierarchical porous carbons for supercapacitors using biochars from different carbonization temperature as catalysts

Chaozheng Liu,[abc] Weimin Chen,[ab] Meichun Li,[abc] Shu Hong,[ab] Wanzhao Li,[ab] Mingzhu Pan,[ab] Qinglin Wu [c] and Changtong Mei *[ab]

[a] *College of Materials Science and Engineering, Nanjing Forestry University, Nanjing 210037, China. E-mail: mei@njfu.edu.cn; Tel: +86-25-5427742.*

[b] *Jiangsu Engineering Research Center of Fast-growing Trees and Agri-fiber Materials, Nanjing 210037, China*

[c] *School of Renewable Natural Resources, Louisiana State University, Baton Rouge, LA 70803, USA*

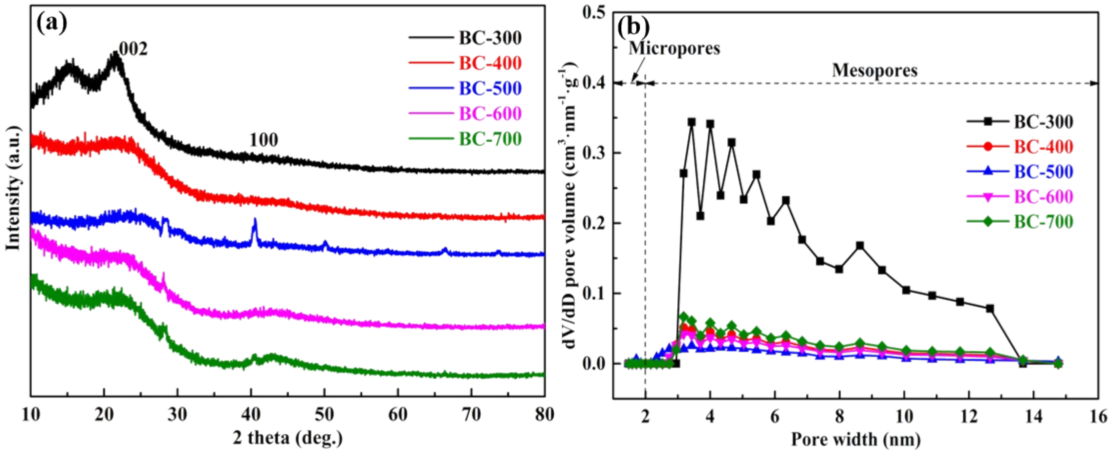

Fig. S1 XRD patterns (a) and pore size distribution (b) of the BCs.

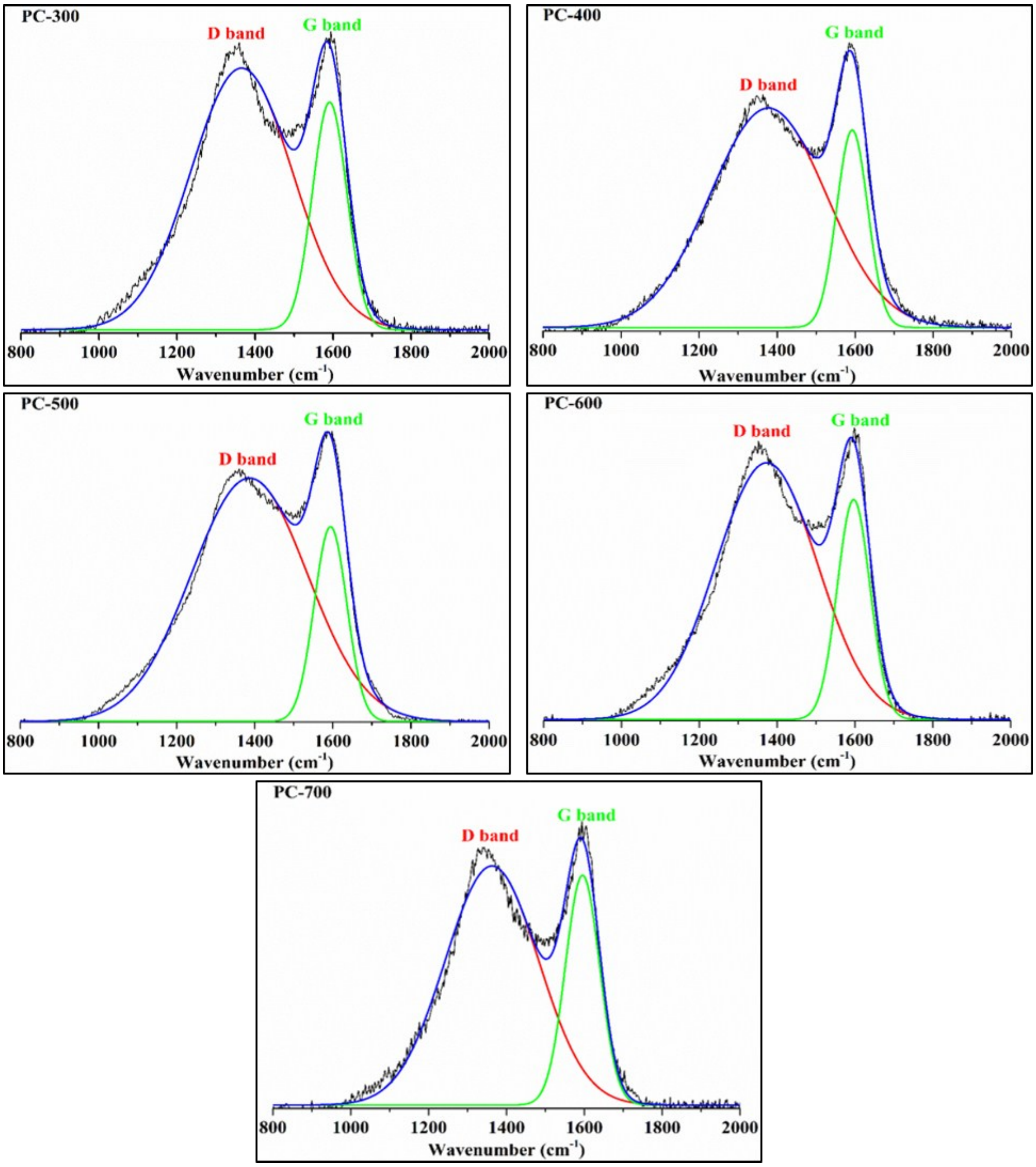

Fig. S2 Fitted Raman spectra of all PCs

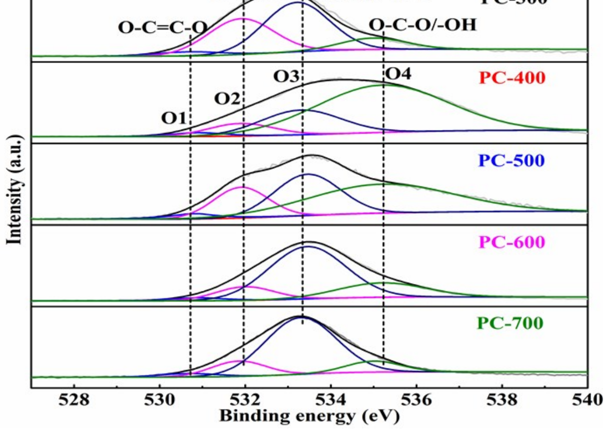

Fig. S3 XPS spectra of O1s for all PCs.

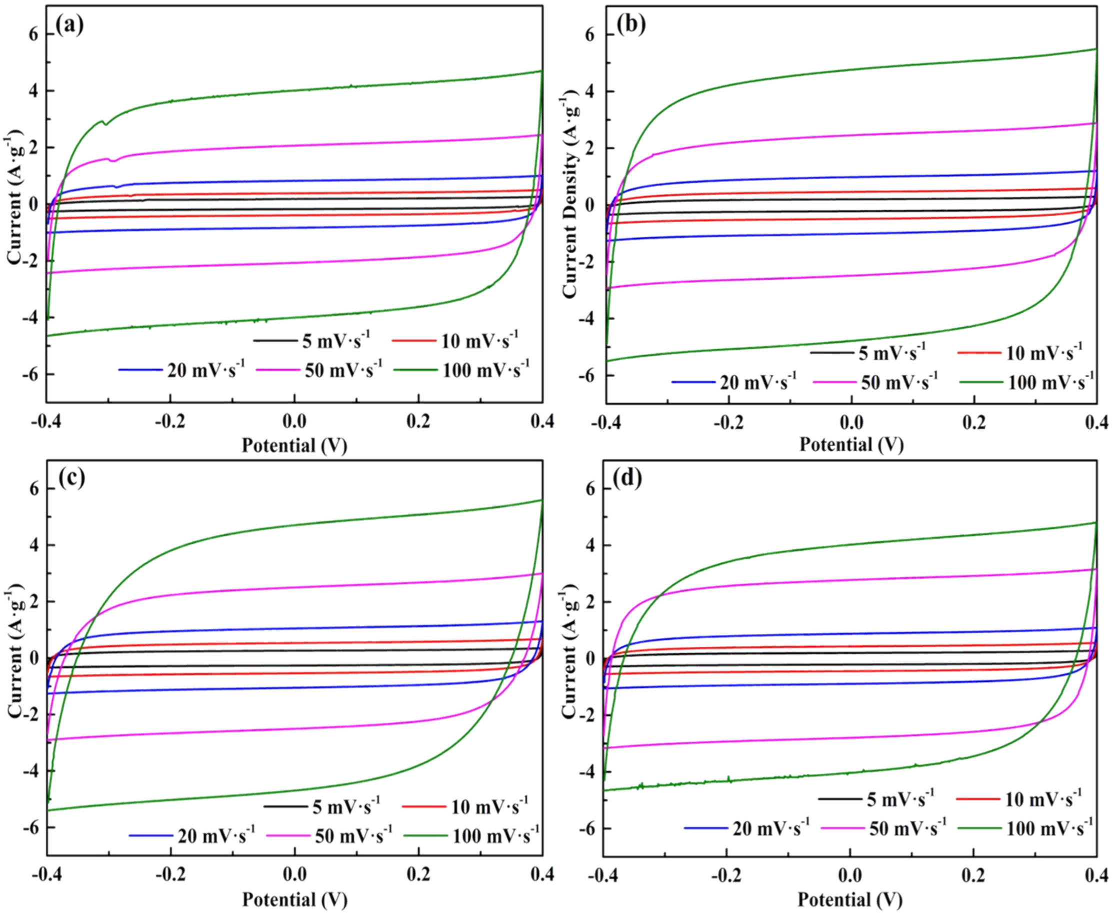

Fig. S4 CV curves of the PC-400 (a), PC-500 (b), PC-600 (c) and PC-700 (d) at different scan rates from 5 to 100 mV·s^{-1} in a voltage range from -0.4 V to 0.4 V.

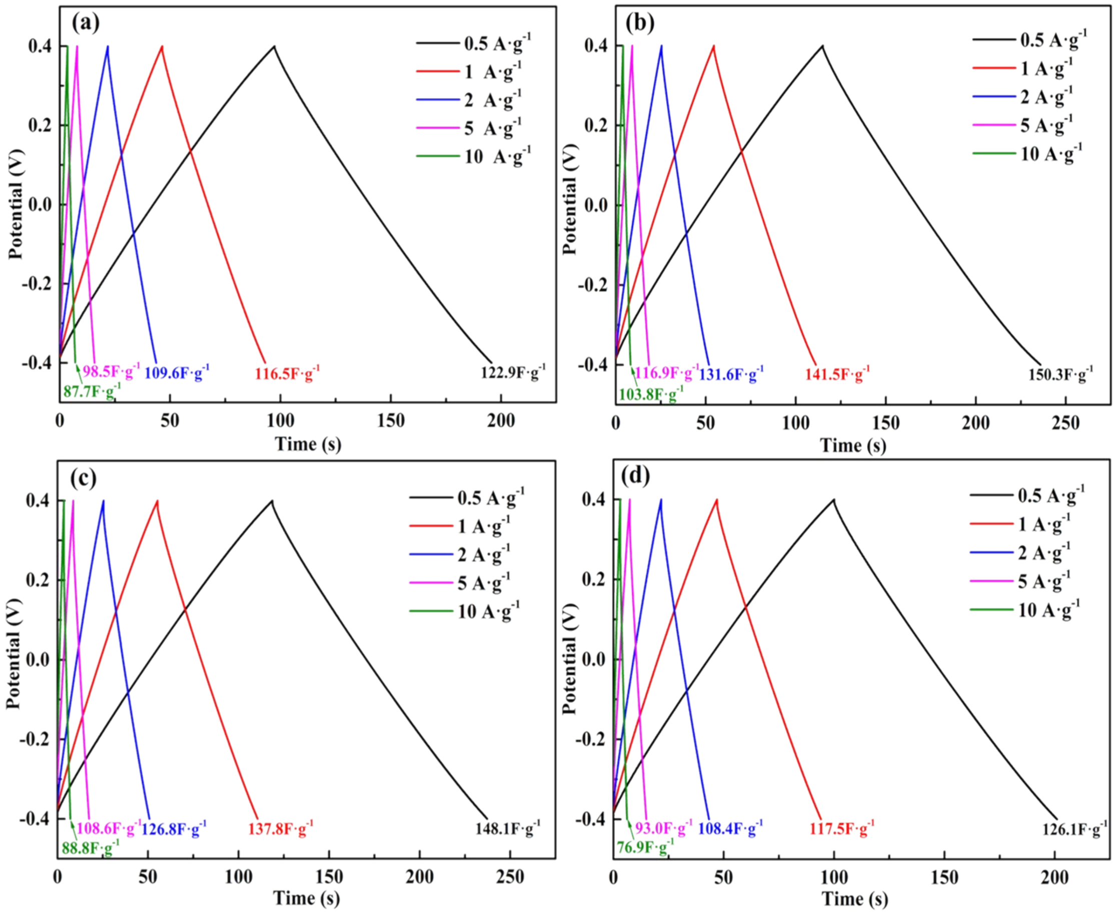

Fig. S5 GCD curves of the PC-400 (a), PC-500 (b), PC-600 (c) and PC-700 (d) at various current densities from 0.5 to 10 A·g^{-1} in a voltage range from -0.4 V to 0.4 V.

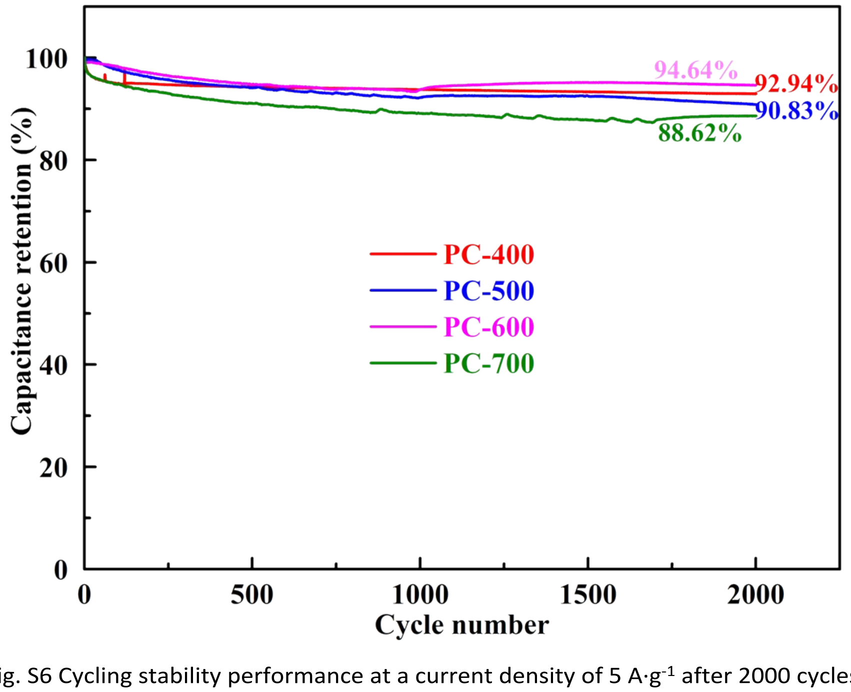

Fig. S6 Cycling stability performance at a current density of 5 A·g^{-1} after 2000 cycles.
